# Medical College of Memphis

**Published:** 1846-05

**Authors:** 


### MEDICAL COLLEGE OF MEMPHIS.

From the Memphis newspapers we learn that the projected medical college of that city is getting under way. The trustees have made the following appointments: J. M. Bybee, M.D., to be professor of Anatomy; D. J. M. Doyle, M.D., professor of Surgery; A. Hopton, M.D., professor of Chemistry and Pharmacy; G. R. Grant, M.D., professor of Theory and Practice. The board advertise for professors to take the chairs of Institutes and Medical Jurisprudence, Materia Medica, and Obstetrics; application to be made to the Secretary of the Board before the third day of July. It is promised that "the names of the successful candidates only will be made public."

